# Enhanced early detection of thyroid cancer using ensemble machine learning and serum proteomics

**DOI:** 10.3389/fonc.2026.1807894

**Published:** 2026-03-25

**Authors:** Da Zhang, Jiangbo Ding, Zhangjian Zhou

**Affiliations:** 1 Xi’an Jiaotong University, Xi’an, China; 2 Department of Clinical Research, Xianyang Hospital of Yan’an University, Xianyang, China; 3 The Comprehensive Breast Care Center, The Second Affiliated Hospital of Xi’an Jiaotong University, Xi’an, China

**Keywords:** early diagnosis, machine learning, MALDI-TOF mass spectrometry, serum proteomic profiling, thyroid carcinoma

## Abstract

**Background:**

Thyroid cancer presents a significant clinical challenge due to asymptomatic onset and poor post-metastasis prognosis. Current imaging methods lack specificity, and single biomarkers show limited diagnostic accuracy. This study aimed to develop and validate a diagnostic model integrating serum proteomics and machine learning for early detection.

**Methods:**

Serum samples from 414 thyroid cancer patients and 430 healthy controls were analyzed using MALDI-TOF MS. Multiple machine learning algorithms were applied to construct diagnostic models and evaluated in an independent test set. Model interpretability was assessed using SHAP and LIME, and key peptide were identified through feature importance analysis. A simplified diagnostic model was subsequently reconstructed using the selected features. Discriminative performance was evaluated using ROC-AUC and DCA. GO and KEGG enrichment analyses were performed to elucidate the biological functions of differentially expressed proteins.

**Results:**

The integrated machine learning model demonstrated excellent discriminative performance. Interpretability analyses indicated that the high performance of the model was driven by the robust and coordinated contributions of multiple features. 12 peptide peaks significantly associated with thyroid cancer were identified, and the simplified model based on these features maintained high diagnostic accuracy and provided greater net clinical benefit than single-protein biomarkers. Enrichment analysis revealed that those proteins were involved in immune regulation, lipid metabolism, and other cancer-related biological processes.

**Conclusions:**

This study established and validated a serum peptide-based diagnostic model integrating machine learning for thyroid cancer, exhibiting promising diagnostic performance in the single-center cohort, providing a non-invasive strategy for early detection and a basis for further research.

## 1 Introduction

Thyroid carcinoma (TC) is the most prevalent malignant neoplasm of the endocrine system, with its incidence steadily increasing worldwide in recent years (1). Based on histological origin and differentiation status, thyroid cancer is primarily classified into three major categories: differentiated thyroid carcinoma (DTC), medullary thyroid carcinoma (MTC), and anaplastic thyroid carcinoma (ATC) (2, 3). Among these, DTC accounts for approximately 95% of cases and generally carries a favorable prognosis; however, the risk of mortality significantly rises upon the occurrence of invasion, metastasis, or radioiodine (RAI) resistance (4, 5). Although ATC represents only a small proportion of cases, it is highly aggressive, often presents with metastasis at diagnosis, exhibits poor response to radiotherapy and chemotherapy, and has a median survival of merely a few months, rendering it the subtype with the worst prognosis (6). Currently, diagnostic evaluation of thyroid cancer primarily relies on medical imaging in combination with fine-needle aspiration biopsy for cytological assessment and initial subtype classification (7). For patients with early-stage or localized DTC, surgical resection followed by radioactive iodine therapy and thyroid-stimulating hormone suppression remains the standard treatment regimen (8). However, therapeutic options become considerably limited for patients with advanced or metastatic DTC, particularly after the development of RAI resistance, necessitating a shift toward systemic therapies represented by tyrosine kinase inhibitors (TKIs) (9). Meanwhile, due to their rapid progression and highly aggressive biological behavior, ATC and advanced MTC often require prompt initiation of comprehensive treatment strategies centered on TKIs or targeted therapies upon diagnosis, although overall efficacy remains suboptimal (10).

The precise identification of biomarkers closely associated with the pathogenesis and progression of thyroid cancer holds significant importance for early recognition of high-risk patients, implementation of stratified disease management, and dynamic monitoring of therapeutic response. In recent years, rapid advances in molecular biology and immunology have greatly propelled the exploration of biomarkers related to thyroid cancer. For instance, claudin-16 (CLDN16) has been proposed as a potential diagnostic marker for PTC (11), while TG, Ctn, CEA, and Pct are vital for diagnosing and monitoring thyroid cancer (7, 12). These studies offer new perspectives for clinical diagnosis, risk stratification, and treatment monitoring. Nevertheless, constrained by tumor heterogeneity and the complexity of disease progression, there remains a lack of a single biomarker with sufficient sensitivity and specificity to serve as a reliable gold standard for the diagnosis and prognosis evaluation, underscoring an urgent need to identify more stable and reproducible molecular indicators.

Serum is readily accessible and contains abundant proteomic information that reflects both physiological and pathological alterations in the body, rendering it a valuable source for disease biomarker discovery (13). In recent years, the integration of artificial intelligence (AI)–assisted analytical strategies has emerged as a powerful framework for biomarker screening and validation (14–16). AI algorithms, particularly machine learning (ML) and deep learning models, are well suited to uncover complex patterns and nonlinear relationships within high-dimensional data, thereby overcoming the inherent limitations of conventional statistical approaches in feature extraction and predictive performance (17). In this context, the integration of multi-omics data with AI-driven feature selection and predictive modeling holds substantial promise for identifying robust biomarkers with enhanced sensitivity, specificity, and clinical translational potential. Proteomics technologies enable high-throughput profiling of large numbers of proteins, providing a systematic means to characterize disease-related molecular signatures (18). Accordingly, the development of serum protein–based biomarkers offer valuable opportunities for early diagnosis, refined risk stratification, and dynamic monitoring, thereby supporting personalized therapeutic decision-making in thyroid cancer.

In this study, serum samples were collected from patients with thyroid cancer and matched control subjects, followed by comprehensive serum proteomic analysis for systematic peptide peak identification. After data preprocessing and normalization, diagnostic models were constructed using multiple machine learning algorithms and evaluated in an independent test set. To enhance model interpretability and feature robustness, explainable AI approaches were applied to jointly identify peptide features with significant contributions to model performance, thereby pinpointing differentially expressed peptides closely associated with thyroid cancer. These key peptides were subsequently subjected to sequence identification, and machine learning models were rebuilt based on the refined peptide feature set to further assess their diagnostic utility. Receiver operating characteristic (ROC) curve analysis combined with decision curve analysis (DCA) was then employed to comprehensively evaluate the association between candidate biomarkers and the clinical diagnosis of thyroid cancer, thereby validating their potential clinical applicability. Finally, Gene Ontology (GO) and Kyoto Encyclopedia of Genes and Genomes (KEGG) pathway analyses were performed to functionally annotate the differentially expressed proteins and elucidate the biological pathways involved. The functional profiles were further compared with the diagnostic performance of individual proteins, providing additional insight of integrating serum proteomics with machine learning for thyroid cancer diagnosis.

## 2 Materials and methods

### 2.1 Clinical samples and ethical approval

A total of 844 serum samples were collected from patients at the Xianyang Hospital of Yan’an University. Among them, there were 414 patients with thyroid cancer (TC) and 430 healthy control (HC) volunteers. The inclusion criteria were patients diagnosed with thyroid cancer based on the results of routine surgical pathology or cytology, and none of the patients had received chemotherapy, radiotherapy, immunotherapy or targeted therapy. Patients with a history of thyroid cancer, those who had received thyroid - related treatment, those with other concurrent malignant tumors, those with severe organ dysfunction, or those unable to cooperate with follow - up were excluded. This study was conducted using the remaining serum samples after clinical testing, ensuring no leakage of patients’ personal information and avoiding repeated blood sampling. All sample donors signed informed consent forms. This study strictly adhered to the ethical guidelines of the Declaration of Helsinki, and the research protocol had been reviewed and approved by the Ethics Committee of the Xianyang Hospital of Yan’an University (YDXY-KY-2024-002).

### 2.2 Collection and preparation of serum samples

After allowing the whole blood specimens to stand at room temperature for 2 hours, centrifuge them at 3500 rpm for 10 minutes at 4 °C. Collect 500 μL of the supernatant from each tube, aliquot it, and store it at -80 °C. Avoid repeated freeze - thaw cycles.

### 2.3 Experiment design and metrics

**Figure 1:**
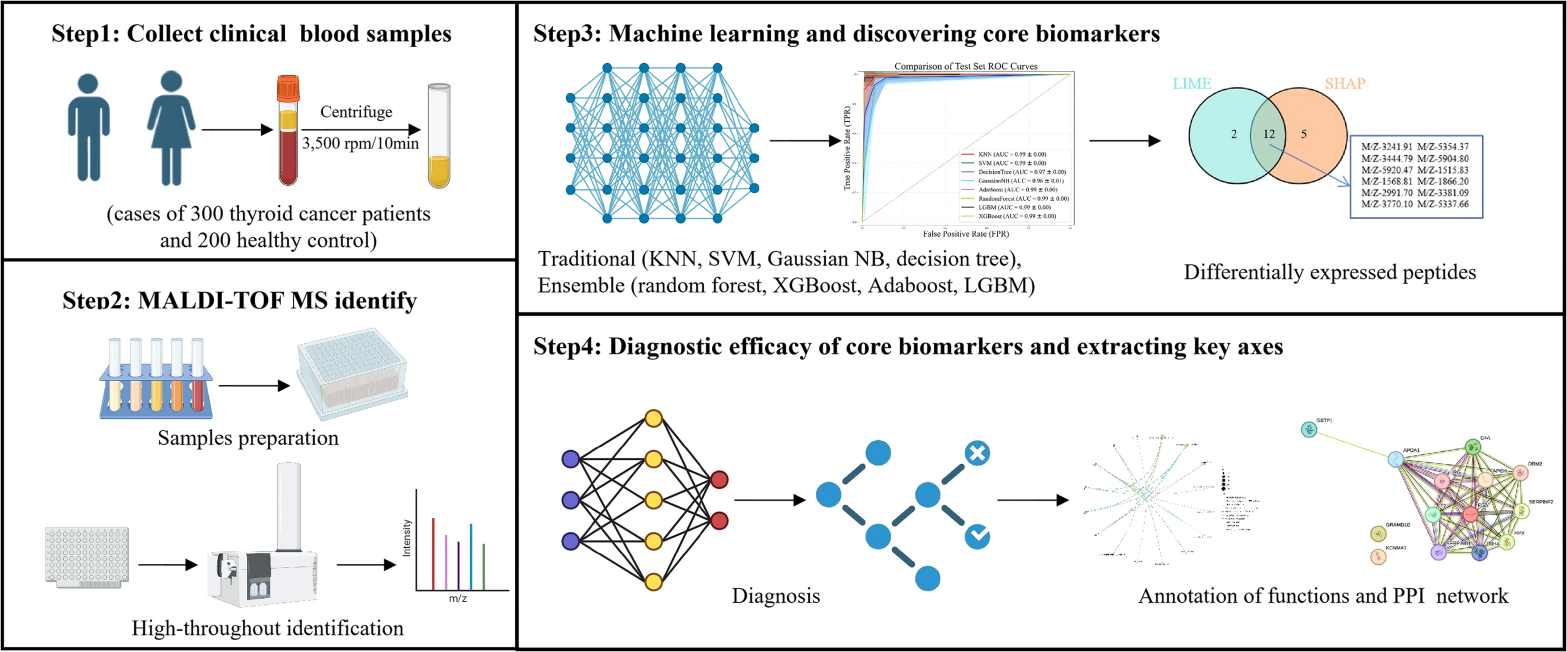
Flowchart of this study.

Serum proteomics analysis was performed using Matrix-Assisted Laser Desorption/Ionization Time-of-Flight Mass Spectrometry (MALDI-TOF/MS). Raw spectral data were preprocessed for peptide peak analysis, normalized to reduce technical variations, and the most significantly differential peptide molecules were screened. Through analysis of 8 machine learning classifiers, high-performance differential peptide segments were identified by extracting feature values, followed by further sequencing via Liquid Chromatography-Electrospray Ionization Tandem Mass Spectrometry (LC-ESI-MS/MS). The workflow of this study was shown in **Figure 1**.

### 2.4 Samples preparation and MALDI-TOF MS analysis

Serum peptide segments were isolated and extracted using the MB-IMAC kit (YTHZ2001, Yuan Tong Huizhe Biotechnology, Xi’an, China) with the following specific steps: 1) Equal volumes of magnetic bead suspension (20 µL) and binding buffer were mixed, followed by the addition of an equal volume of serum sample; the mixture was incubated at room temperature for 5 minutes; 2) The magnetic bead-peptide complex was washed three consecutive times with 100 µL washing buffer, and 10 µL elution buffer was added; 3) A 1 µL aliquot of the purified eluate was mixed with an equal volume of matrix solution, spotted onto a mass spectrometry target plate, and air-dried at room temperature to form co-crystals. MALDI-TOF MS analysis was conducted using the Zybio EXS2600 instrument (Chongqing, China).

### 2.5 Data pre-processing

Raw mass spectrometry data of protein peptide segments from the two sample groups were preprocessed using Python. Total ion current chromatograms were normalized, and the Savitzky-Golay smoothing algorithm was applied to suppress random noise. Adaptive iterative baseline correction was used to subtract background drift, enabling accurate separation of true signals. Finally, spectra before and after processing were visually compared to confirm the authenticity and correspondence of key chromatographic peaks.

### 2.6 Peak feature extraction, normalization, and integrative data matrix construction

The M/Z peaks were detected using the adaptive threshold method, and false positive peaks in the 1500–2000 matrix cluster region were excluded. The iterative greedy algorithm was used to align the cross-sample peaks within a tolerance of 2,500 ppm, correcting for instrument drift and biological variation. Finally, a peak feature matrix with samples as rows and peaks as columns was constructed, with the numerical values being the relative peak areas. Missing values were filled by setting them to zero or by linear interpolation in adjacent intervals.

### 2.7 Machine learning-driven proteomic data analysis

To ensure robust model development and unbiased performance evaluation, the samples were first stratified based on the diagnosis (TC vs. HC) and then randomly partitioned into a training set and an independent test set in a ratio of 8:2. Principal Component Analysis (PCA), Kernel Principal Component Analysis (KPCA), and t-distributed stochastic neighbor embedding (t-SNE) methods were employed to reduce the dimensionality of the features. Eight machine learning models were used for classification analysis, including SVM, KNN, XGBoost, AdaBoost, LGBM, GaussianNB, DecisionTree, and RandomForest. Quantitative evaluation was conducted using AUC-ROC, accuracy, precision, recall, and F1 score to ensure the comprehensiveness and robustness of the conclusions.

Identifying key mass spectrometry features through ensemble learning: Combining random forest and XGBoost to evaluate feature importance based on the Gini index, and integrating Shapley additive explanations (SHAP) and local interpretable model-agnostic explanations (LIME) methods to analyze feature contributions, the top 20 differential peptides were selected as candidate biomarkers. The corresponding peptides and proteins were annotated using tandem mass spectrometry technology, and their relative abundance matrices were constructed.

The biomarker matrix was subjected to unsupervised clustering using the binary k-means and Birch algorithms, and the clustering structure was visualized through Uniform Manifold Approximation and Projection (UMAP) and compared with the original dataset. Furthermore, the clinical discrimination efficacy and practicality of these biomarkers were systematically evaluated using AUC-ROC and DCA. The performance of various machine learning classifiers based on the original data and the annotated biomarker matrix was compared to verify the effectiveness of the selected biomarkers in improving diagnostic performance and model interpretability.

### 2.8 Clinical validation of public data

Candidate biomarkers were validated against the PRIDE database (https://www.ebi.ac.uk/pride/). Data-independent acquisition mass spectrometry analysis was utilized to evaluate the AUC-ROC and DCA values of target proteins.

### 2.9 Biological pathway analysis

GO and KEGG pathway analyses were performed to investigate the biological functions and molecular pathways associated with the differentially expressed proteins. GO analysis annotated proteins in terms of biological process, cellular component, and molecular function, while KEGG analysis was used to identify significantly enriched signaling and metabolic pathways.

### 2.10 Statistical analysis

All computational analyses and graphing were performed in Python 3.7. Data processing, transformation, and matrix construction were carried out using the pandas and NumPy libraries. Data visualization was implemented using Matplotlib and Seaborn.

## 3 Results

### 3.1 Clinical information

A total of 414 patients with thyroid cancer and 430 healthy controls were enrolled. The median age of the two groups was 48 (35, 58) years and 48 (42, 55) years, respectively. T test showed that there was no significant difference in age distribution between the two groups (*P* = 0.788, values with *P*>0.05 were defined as lacking statistical significance).

### 3.2 Mass spectrometry data and differential peptides

**Figure 2:**
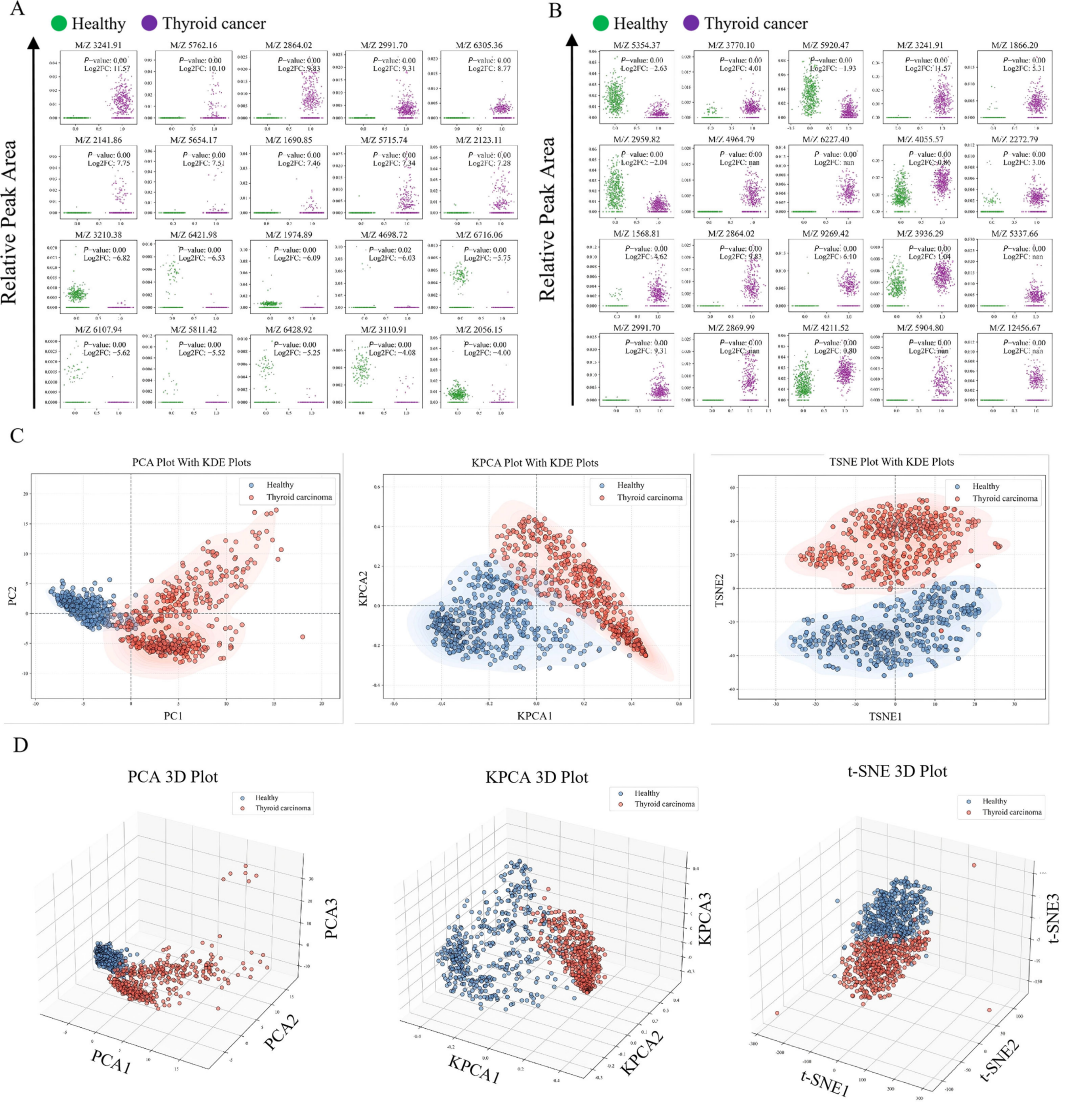
Mass spectrometry data and screening of differential peptides. **(A)** The 20 peptide segments with the greatest expression differences (log_2_FC). **(B)** The 20 peptide segments with the greatest statistical differences (p-value). **(C, D)** After dimensionality reduction using Principal Component Analysis (PCA), Kernel Principal Component Analysis (KPCA), and t-SNE, the 2D/3D visualization of the mass spectrometry data is presented. The shaded area represents the distribution area of the samples, visually demonstrating their spatial dispersion.

The serum peptide segments of thyroid cancer patients and healthy donors were analyzed using MALDI-TOF/MS, and characteristic property spectra within the mass range of 1000-10,000 KDa were obtained. Each experiment was repeated three times. After fitting and normalization processing of the original data, the mass spectral peaks showed an overlapping distribution pattern. To evaluate its diagnostic potential, the top 20 peptide segments with the most significant expression differences (**Figure 2A**) and the top 20 peptide segments with the most significant statistical significance (**Figure 2B**) were selected for subsequent analysis. Further, PCA, KPCA, and t-SNE were used to reduce the dimension of the mass spectral data and achieve two-dimensional and three-dimensional visualization, which intuitively presented the distribution characteristics of different samples in the multi-dimensional space (**Figures 2C, D**).

### 3.3 Evaluation of diagnostic efficiency of thyroid cancer diagnostic models based on eight ML algorithms

**Figure 3:**
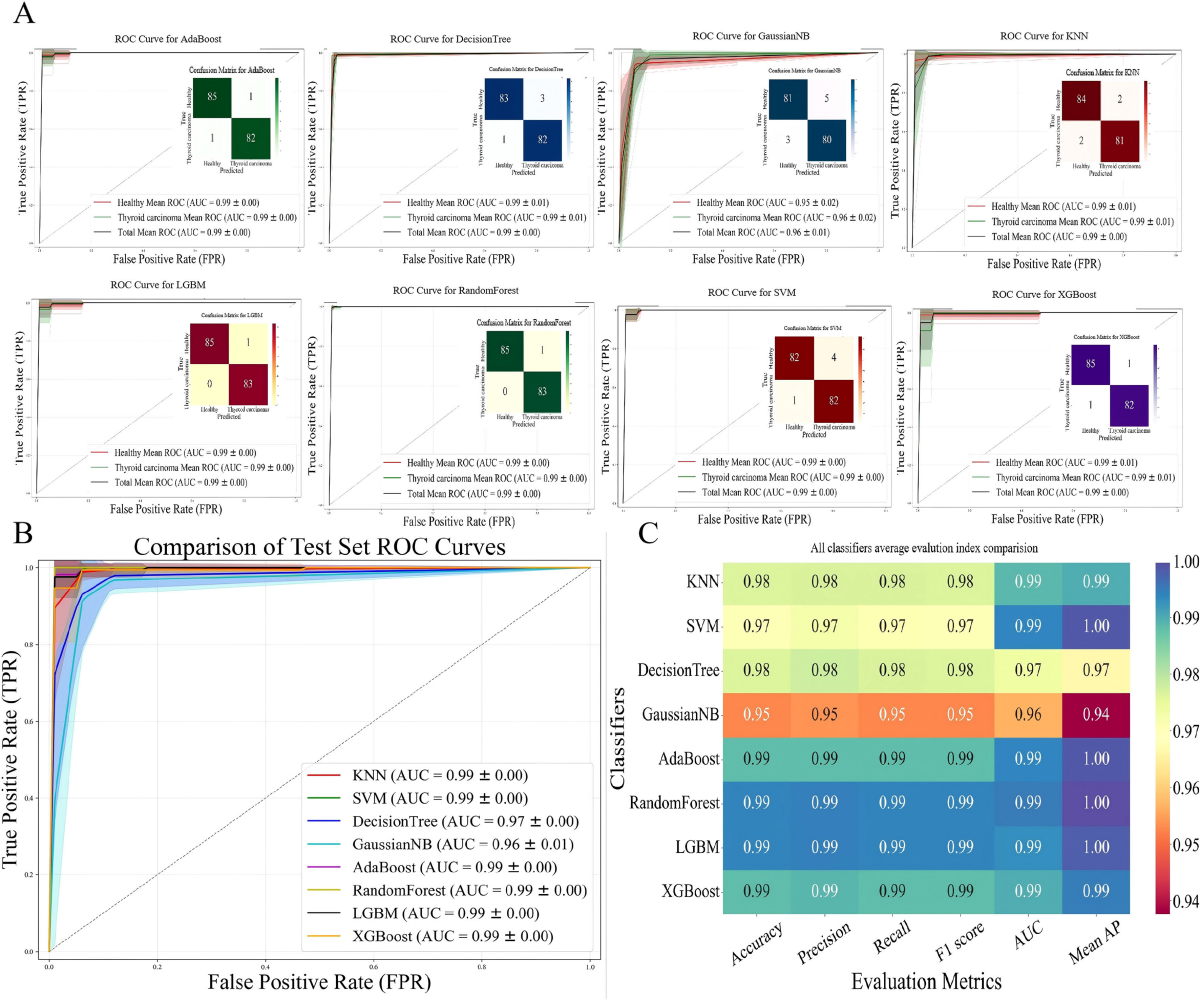
Evaluation of diagnostic efficacy using different machine learning algorithm. **(A)** ROC curves and confusion matrices for classifying two groups of differential peptide peaks using eight different machine learning algorithms. The shaded areas represent the variability of performance indicators across cross-validation iterations (confidence intervals), indicating the robustness of the model. The confusion matrix quantifies the classification results: the TP (top left) and TN (bottom right) on the main diagonal represent correct predictions, while the FP and FN on the off-diagonals represent incorrect classifications. **(B)** Integrals of different ROC curves and their relationships with TPR and FPR. **(C)** Performance indicators of different machine learning algorithms, including accuracy, precision, recall, and F1 score.

This study divided the thyroid cancer dataset into a 20% test set and an 80% training set. ROC curves and confusion matrices were used to systematically evaluate the classification performance of 8 machine learning models. The results showed that the AdaBoost, DecisionTree, KNN, LGBM, RandomForest, SVM, and XGBoost models performed exceptionally well, with an AUC of 0.99 ± 0.00 for each, and the confusion matrices also indicated extremely high prediction accuracy of the models; only the GaussianNB model had a relatively lower AUC of 0.96 ± 0.01 (**Figure 3A**). These results indicate that the features of this dataset are strongly correlated with the classification labels of thyroid cancer, and most of the testing models can achieve efficient classification.

Furthermore, by plotting the ROC curve comparison diagrams of the eight models on the test set, the discriminative ability of each model was intuitively demonstrated from the perspectives of true positive rate and false positive rate. The dataset features and classification labels have a high degree of correlation, and most models in this task exhibit superior classification performance (**Figure 3B**). This study further created a multi-index evaluation heatmap based on accuracy, precision, recall, F1 score, AUC, and mean average precision (**Figure 3C**). The results showed that the AdaBoost, LGBM, RandomForest, and XGBoost models achieved 0.99-1.00 in all indicators, demonstrating strong classification stability and accuracy; the KNN (0.98-0.99) and SVM (0.97-1.00) models had various indicators at a relatively high level; while the DecisionTree (0.97-0.98) and GaussianNB (0.94-0.96) models performed relatively weakly, with GaussianNB being the lowest in all indicators.

### 3.4 Feature selection in different machine learning models

**Figure 4:**
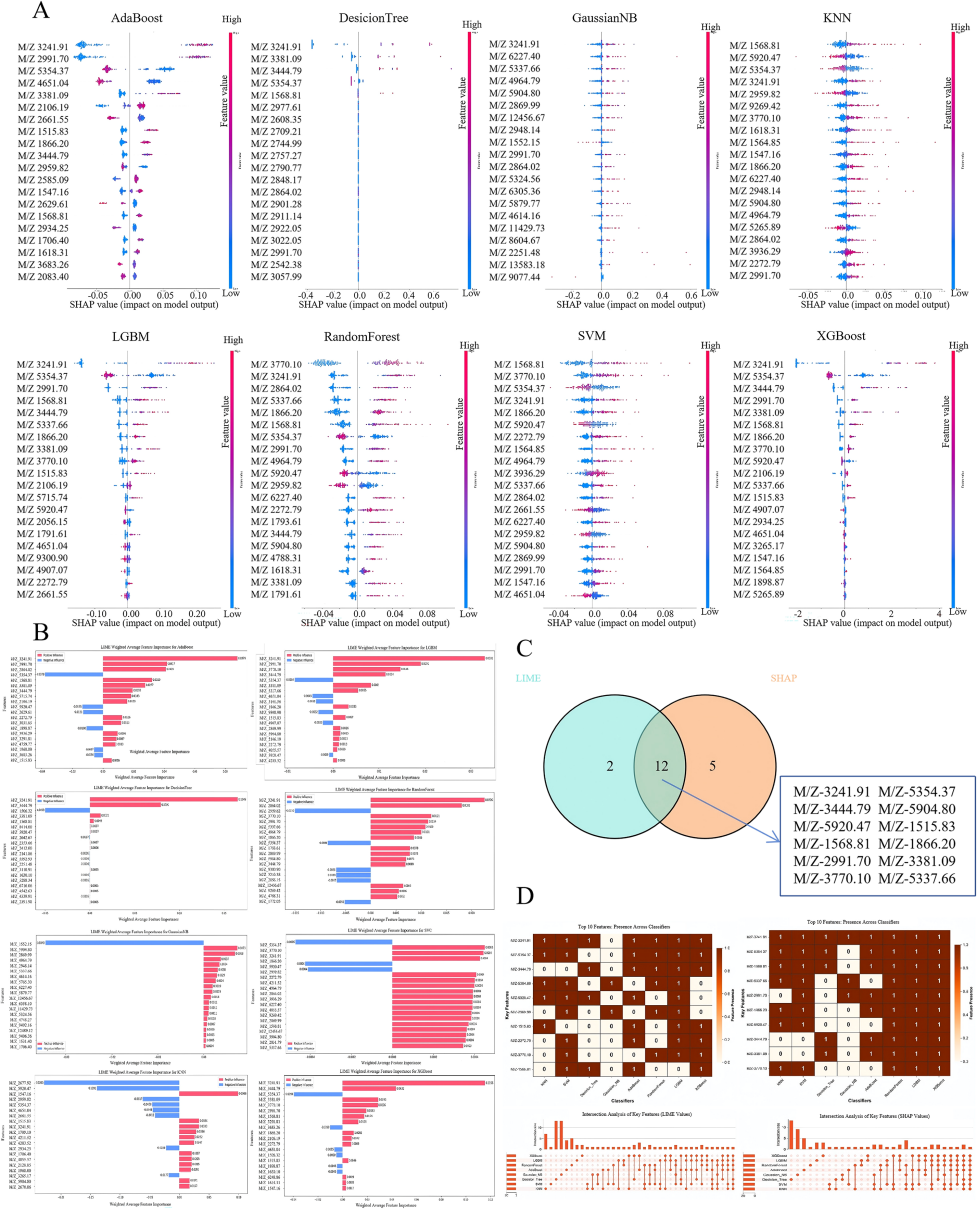
Feature selection in different machine learning models. **(A, B)** The highly performing differential peptide segments identified through the extraction of features using different machine learning algorithms. **(C)** Key prediction features. **(D)** Shapley analysis and **(E)** LIME analysis for different machine learning algorithms.

To analyze the decision-making mechanism of the model and verify the robustness of key predictive features, this study integrated LIME, SHAP, and feature correlation analysis to conduct interpretability evaluations on 8 machine learning models. The global contribution analysis of SHAP revealed that the core feature contributions of AdaBoost, LGBM, RandomForest, and XGBoost models were highly heterogeneous, and the SHAP values of some features fluctuated strongly (such as M/Z = 5904.80, 5920.47, 5354.37, 5337.66, 3770.10, 3444.79, 3381.09, 3241.91, 2991.70, 1866.20, 1568.81, 1515.83), suggesting that these features might be key factors for thyroid cancer prediction; while the feature contribution distribution of the GaussianNB model was relatively concentrated, which was consistent with its weak classification performance (**Figure 4A**). The local importance analysis of LIME indicated that the core positive contribution features of LGBM and XGBoost were consistent, while the feature importance ranking of DecisionTree was significantly different, reflecting the heterogeneity of local decision logic of different models (**Figure 4B**). The Venn diagram showed that the 2 interpretation methods shared 12 core features, verifying the cross-method robustness of key predictive features; moreover, LIME had 2 unique features and SHAP had 5 unique features, which reflected the differences in the emphasis of global and local explanations (**Figure 4C**). The feature correlation heatmap and interaction analysis revealed that some features had strong collinearity, and the combined effect of specific feature pairs was significantly greater than the individual effect, providing potential targets for subsequent exploration of the biological mechanism of thyroid cancer occurrence (**Figure 4D**). Subsequent LC-ESI-MS/MS sequencing and UniProt annotation mapped these peptide peaks to eight corresponding proteins (**Table 1**). In summary, this study conducted multi-dimensional interpretability analysis to verify the reliability of model performance, clarified the key predictive features and their interactions that drive thyroid cancer classification prediction, made the model decision process transparent, and provided data support for subsequent functional verification.

**Table 1:** Results of differential peptide identification.

Mass(Da)	Annotated sequence	Length	Positions in master proteins	Gene names	Protein names
3241.91	NRGDSTFESKSYKMADEAGSEADHEGTHST	30	P02671	FGA	Fibrinogen alpha chain
3444.79	SETESRGSESGIFTNTKESSSHHPGIAEFPSR	32	P02671	FGA	Fibrinogen alpha chain
5904.80	SSSSSSSSSSSSSSSVHEPKMDALIIPVTMEVPCDSRGQRMWWAFLASSMVTFF	54	Q12791	KCNMA1	Calcium-activated potassium channel subunit alpha-1
5920.47	SCVLVLLVILNMMLFYKLWMLEYTTQTLTAWQGLRLQERLPQSQTEWAQ	53	Q3KR37	GRAMD1B	Protein Aster-B
1515.83	[P].TSAHGNVAEGETKPD.[P]	15	P02790	HPX	Hemopexin
1568.81	[G].FKSHALQLNNRQI.[R]	13	P0C0L4	C4A	Complement C4-A
1866.20	[L].FEKKSLEDKTERELL.[E]	15	P00734	F2	Prothrombin
2991.70	R.MLLADQGQSWKEEVVTVETWQEGSLK.A	26	P09211	GSTP1	Glutathione S-transferase P
3381.09	LATVYVDVLKDSGRDYVSQFEGSALGKQLNL	31	P02647	APOA1	Apolipoprotein A-I
3770.10	A.GAAGSRMNFRPGVLSSRQLGLPGPPDVPDHAAYHPF.R	36	B2RMS9	ITIH4	Inter-alpha (Globulin) inhibitor H4 (Plasma Kallikrein-sensitive glycoprotein)
5337.66	[K].SSSYSKQFTSSTSYNRGDSTFESKSYKMADEAGSEADHEGTHSTKRGHA.[K]	49	P02671	FGA	Fibrinogen alpha chain

### 3.5 Validation of thyroid cancer diagnostic models and diagnostic efficacy of core biomarkers

**Figure 5:**
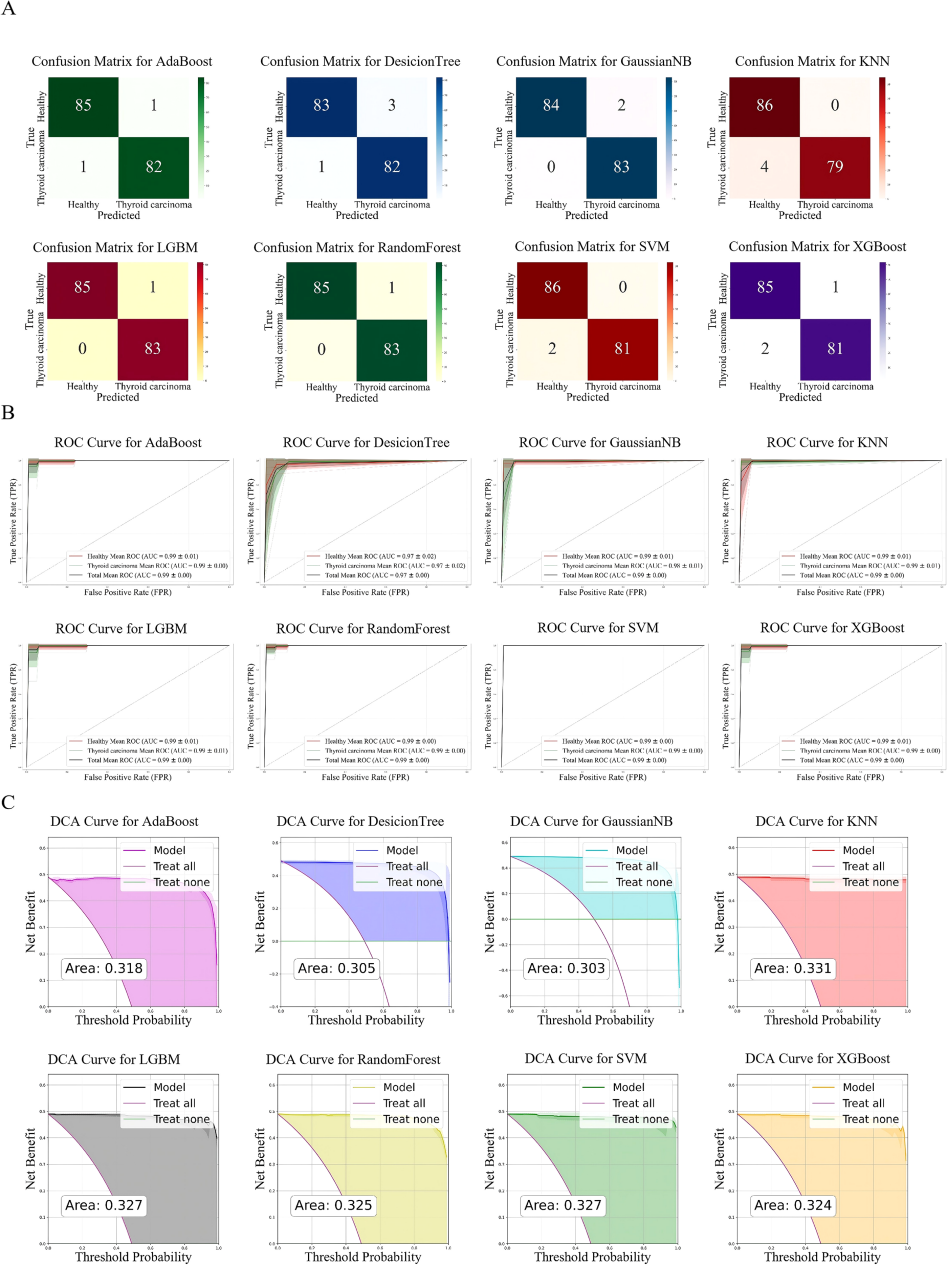
Evaluation of the diagnostic efficacy of differential peptides. **(A)** Confusion matrices illustrating the classification performance of different machine learning algorithms. **(B)** ROC curves and corresponding AUC values for differential peptides under different machine learning algorithms. **(C)** DCA evaluating the clinical net benefit of the machine learning models.

Based on the screened and identified differentially expressed proteins, we reconstructed multiple machine learning models using an unsupervised classification strategy to evaluate the potential diagnostic value of these characteristic proteins for thyroid cancer. As shown in **Figure 5A**, the classification performance of different algorithms is presented through confusion matrices. The diagnostic capability of each model was further assessed using ROC-AUC values. The mean AUC values under different algorithms were as follows: for AdaBoost, Total mean AUC = 0.99 ± 0.00 (Healthy mean AUC = 0.99 ± 0.01, Thyroid carcinoma mean AUC = 0.99 ± 0.00), for Decision Tree, Total mean AUC = 0.97 ± 0.00 (Healthy mean AUC = 0.97 ± 0.02, Thyroid carcinoma mean AUC = 0.97 ± 0.02), for GaussianNB, Total mean AUC = 0.99 ± 0.00 (Healthy mean AUC = 0.99 ± 0.01, Thyroid carcinoma mean AUC = 0.98 ± 0.01), for KNN, Total mean AUC = 0.99 ± 0.00 (Healthy mean AUC = 0.99 ± 0.01, Thyroid carcinoma mean AUC = 0.99 ± 0.01), for LGBM, Total mean AUC = 0.99 ± 0.00 (Healthy mean AUC = 0.99 ± 0.01, Thyroid carcinoma mean AUC = 0.99 ± 0.01), for Random Forest, Total mean AUC = 0.99 ± 0.00 (Healthy mean AUC = 0.99 ± 0.00, Thyroid carcinoma mean AUC = 0.99 ± 0.00), for SVM, Total mean AUC = 0.99 ± 0.00 (Healthy mean AUC = 0.99 ± 0.00, Thyroid carcinoma mean AUC = 0.99 ± 0.00), for XGBoost, Total mean AUC = 0.99 ± 0.00 (Healthy mean AUC = 0.99 ± 0.00, Thyroid carcinoma mean AUC = 0.99 ± 0.00) (**Figure 5B**). The results indicate that all algorithms demonstrated favorable discriminative performance, with their average AUC values remaining at a relatively high level. Among them, the GaussianNB model constructed based on the differential features showed particularly changed in disease discrimination, highlighting its sensitivity to the selected features. Furthermore, DCA was used to evaluate the potential clinical application value of the models in decision-making. The results showed that the net benefit curves of all models were above the baseline reference line, with corresponding area values of 0.318, 0.305, 0.303, 0.331, 0.327, 0.325, 0.327, and 0.324, respectively (**Figure 5C**). This indicates that the machine learning models based on differentially expressed proteins possess good clinical diagnostic efficacy across a wide range of threshold probabilities. In summary, the machine learning strategy integrating multiple features demonstrates clear advantages in the auxiliary diagnosis of thyroid cancer.

### 3.6 Diagnostic evaluation of differentially expressed peptides

**Figure 6:**
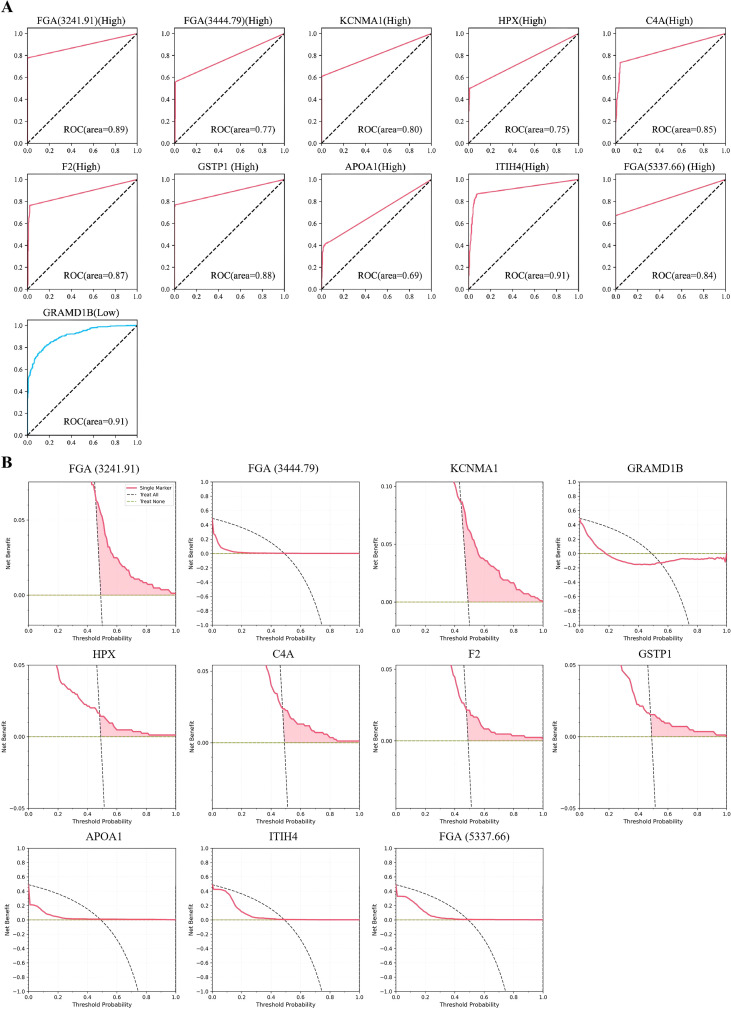
Diagnostic performance of individual characteristic proteins. **(A)** ROC curve analysis assessing the diagnostic accuracy of individual proteins based on PRIDE database validation. **(B)** DCA evaluating the clinical net benefit of single biomarkers.

To further evaluate the individual diagnostic potential of the selected characteristic proteins, we examined the expression levels of these molecules using the PRIDE database and independently assessed their performance as single biomarkers for thyroid cancer. ROC analysis revealed that the AUC values for FGA(3241.91), FGA(3444.79), KCNMA1, HPX, C4A, F2, GSTP1, APOA1, ITIH4, FGA(5337.66), and GRAMD1B were 0.89, 0.77, 0.80, 0.75, 0.85, 0.87, 0.88, 0.69, 0.91, 0.84, and 0.91, respectively(**Figure 6A**), indicating that several proteins possess moderate to strong discriminative ability for distinguishing thyroid cancer patients from healthy controls. In addition, DCA revealed that only FGA(3241.91), KCNMA1, HPX, C4A, F2, and GSTP1 demonstrated net benefit curves above the baseline reference line, but even for the best-performing marker, KCNMA1, its maximum net benefit value was merely 0.0618(**Figure 6B**). These results indicate that, although certain proteins show some diagnostic value at the statistical level, the clinical utility of individual biomarkers in decision-making remains limited, highlighting the constrained diagnostic efficacy of single indicators and underscoring the importance of multi-marker integrated analysis.

### 3.7 GO and KEGG pathway analyses

**Figure 7:**
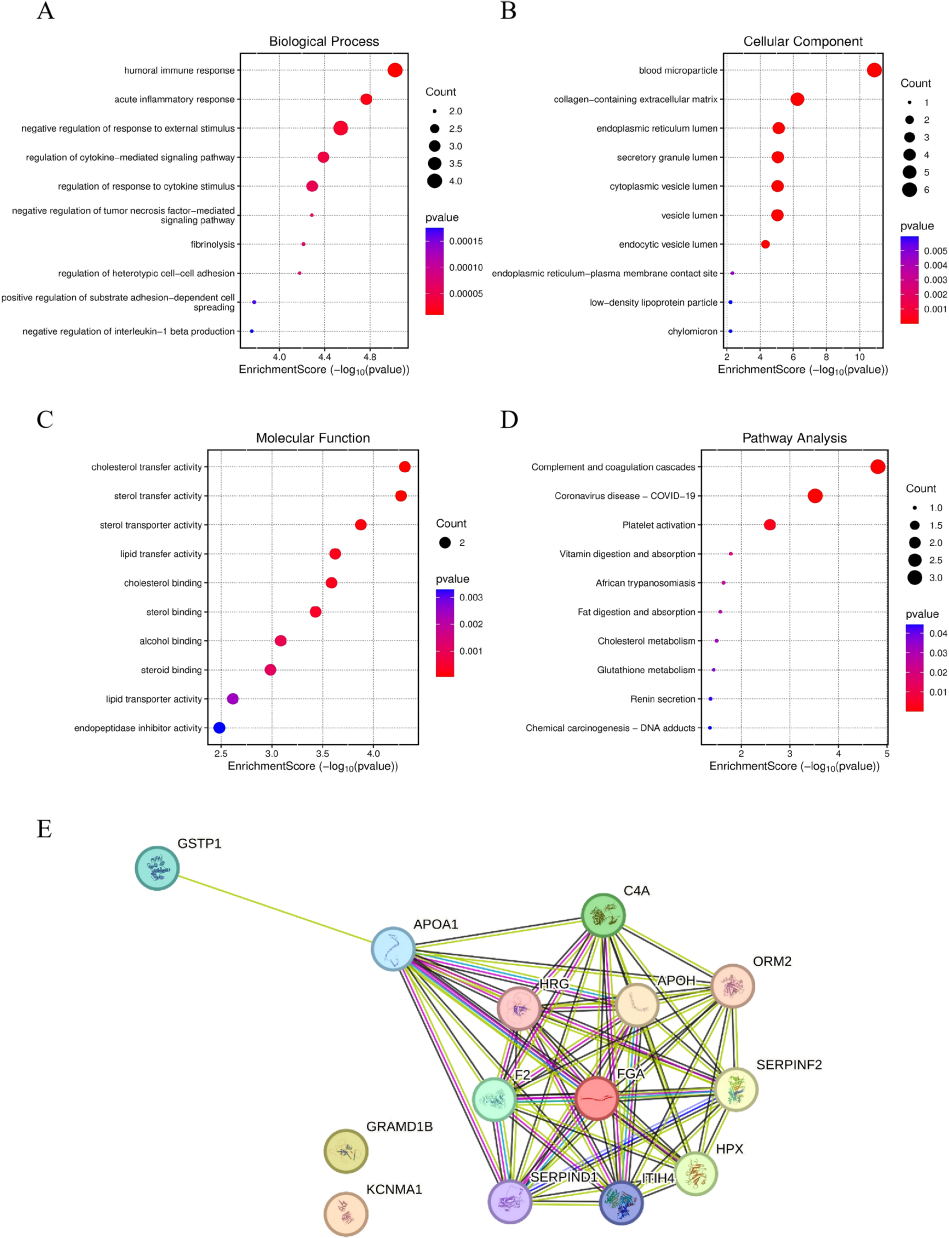
Functional annotation of the differentially expressed proteins. **(A)** GO enrichment analyses of biological processes, **(B)** GO enrichment analyses of cellular components, **(C)** GO enrichment analyses of molecular functions, **(D)** KEGG pathway enrichment analysis, **(E)** Protein–protein interaction network generated from the STRING database.

To systematically elucidate the potential biological functions of the aforementioned 10 differentially expressed proteins, we further conducted GO and KEGG pathway enrichment analyses. As shown in **Figure 7A**, biological process analysis revealed that these proteins were primarily enriched in humoral immune response, negative regulation of response to external stimulus, and regulation of cytokine-mediated signaling pathways, suggesting their potential involvement in tumor-related immunomodulatory processes. At the cellular component level, these molecules were mainly localized to blood microparticles, collagen-containing extracellular matrix, and the endoplasmic reticulum lumen (**Figure 7B**), indicating their close association with secretory processes and the extracellular microenvironment. Molecular Function analysis further demonstrated significant enrichment in cholesterol transporter activity, lipid transporter activity, and sterol transporter activity (**Figure 7C**), implying that lipid metabolism remodeling may play an important role in the development and progression of thyroid cancer. Moreover, KEGG pathway analysis further indicated that these differentially expressed proteins were mainly involved in pathways such as complement and coagulation cascades, platelet activation, and vitamin digestion and absorption (**Figure 7D**). Additionally, the protein–protein interaction network constructed based on the STRING database showed that these molecules are functionally interconnected, forming an interaction module centered on coagulation- and immune-related proteins (**Figure 7E**). Integrating the above analytical results, these differentially expressed proteins appear to act synergistically in various biological processes such as immune regulation and lipid metabolism, potentially collectively contributing to the initiation and progression of thyroid cancer.

## 4 Discussion

Blood harbors a complex spectrum of biomolecules, including proteins, cytokines, and metabolites, which collectively reflect systemic physiological and pathological alterations (19). Increasing evidence suggests that quantitative assessment of circulating biomolecules provides valuable insights into disease presence and progression across diverse conditions, such as autoimmune disorders, cardiovascular diseases, and malignancies (20–22). In the context of thyroid cancer, serum-based analysis offers distinct advantages, including minimal invasiveness, cost-effectiveness, and high reproducibility, making it particularly suitable for repeated measurements and large-scale screening in high-risk populations. Serum proteins function as key mediators of systemic homeostasis and can serve as integrated molecular readouts of tumor-associated biological remodeling rather than isolated tumor-derived signals (23). Despite these advantages, the clinical translation of serum biomarkers for early thyroid cancer detection has been hindered by the limited specificity of imaging-based assessments and the suboptimal performance of individual protein markers (24, 25). To addressed it, we combined MALDI-TOF MS-based serum peptidomics analysis with multiple algorithms driven machine learning to identify serum-derived protein markers that can provide a biologically and clinically meaningful framework for non-invasive thyroid cancer assessment.

In this study, we constructed machine learning models based on eight different algorithms and identified differentially expressed peptides closely associated with thyroid cancer through an intersectional feature selection strategy integrating SHAP and LIME analyses. These peptides include FGA (3241.91), FGA (3444.79), KCNMA1, HPX, C4A, F2, GSTP1, APOA1, ITIH4, FGA (5337.66), and GRAMD1B Subsequently, machine learning models built on these differentially expressed peptides demonstrated excellent performance in disease diagnostic validation. Importantly, comparison with single-protein–based diagnostic strategies revealed a clear distinction between statistical discrimination and clinical utility. Although some individual protein indicators exhibited good discriminatory ability in ROC analysis, DCA revealed their limited clinical net benefit, suggesting that relying solely on statistical discrimination may not translate into practical clinical utility (**Figure 6**). In contrast, machine learning models integrating the above multi−peptide signals not only consistently achieved higher and more stable AUC values but also showed sustained and significant clinical net benefit across a wide range of threshold probabilities. These findings indicate that integrating complementary molecular signals through algorithmic approaches can effectively overcome the inherent variability and biological heterogeneity of individual biomarkers, thereby enhancing the robustness and clinical applicability of diagnostic models.

Functional enrichment analyses revealed that the identified proteins converge on immune regulation, coagulation cascades, and lipid transport pathways (**Figure 7**). The enrichment of humoral immune response and cytokine-mediated signaling pathways suggests an active interplay between tumor development and systemic immune modulation. Concurrently, the involvement of complement and coagulation cascades, as well as platelet activation pathways, aligns with the well-recognized pro-thrombotic state associated with malignancy and its role in tumor progression and metastasis. Moreover, the enrichment of cholesterol and lipid transporter activities highlights metabolic reprogramming as a hallmark of thyroid cancer, potentially influencing membrane dynamics, signaling transduction, and tumor–microenvironment interactions. In addition, protein-protein interaction analysis revealed that these molecules form a tightly connected functional network, which further provided a biological rationale for the superior diagnostic performance of multi-feature machine learning models, as algorithmic integration captures coordinated pathway perturbations that cannot be adequately represented by individual protein markers. Based on existing literature, we further examined the functional and clinical relevance of the proteins identified in this study. Complement component C4A has been reported to exhibit high diagnostic accuracy for papillary thyroid carcinoma with sensitivity and specificity of 91.67% and 83.33%, respectively (26, 27). GSTP1 has been implicated in increased susceptibility to thyroid cancer (28, 29), while APOA1 levels were shown to be negatively correlated with disease occurrence in male PTC patients (30). In addition, ITIH4 has been consistently reported as differentially expressed across multiple solid tumors (31). Although the roles of FGA, KCNMA1, HPX, F2, and GRAMD1B in thyroid cancer remain unclear, these molecules have been shown to participate in tumor-related processes in other malignancies. FGA has been reported to regulate tumor progression through NOTCH3- and FAK/ERK-related pathways (32, 33), KCNMA1 to interact with phosphorylated BRAF in glioma (34), and GRAMD1B to modulate breast cancer cell migration via the JAK/STAT and Akt signaling pathways (35). Collectively, these findings provide biological support for the involvement of the identified proteins in thyroid cancer–associated serum proteomic alterations observed in this study.

From a clinical perspective, the proposed serum proteomics–based machine learning framework offers a non-invasive and scalable strategy for thyroid cancer screening and auxiliary diagnosis. Such an approach may serve as a valuable complement to ultrasonography and fine-needle aspiration, particularly in ambiguous cases or high-risk populations. Furthermore, the integration of proteomic signatures with clinical parameters or imaging features may further enhance diagnostic precision and support individualized clinical decision-making.

This study has certain limitations. Firstly, the samples were all from a single center and were not collected with time stratification. Although strict inclusion and exclusion criteria were used to control confounding factors, the population characteristics of the single-center samples were highly homogeneous, which may limit the model’s universality in different regions and institutions with varying levels of diagnosis. The model’s diagnostic efficacy in different populations and clinical scenarios has not been fully verified yet. Secondly, no independent external cohort validation or robustness assessment based on time stratification was conducted, making it difficult to fully reflect the diagnostic stability of the model in the time dimension and its adaptability to changes in clinical data distribution. This to some extent affects its clinical promotion value. In response to these limitations, subsequent studies will be carried out in the following aspects: Firstly, conducting multi-center prospective studies, combining different regions and grade medical institutions, and including diverse populations of thyroid cancer, to conduct external validation of this model and iteratively optimize it to enhance its cross-population universality; Secondly, conducting robustness assessment of the single-center samples through time stratification design, simulating clinical sequential diagnostic scenarios, analyzing the dynamic changes in model performance and its potential influencing factors, to provide a basis for the long-term application of the model; Thirdly, combining multi-center and time stratification results, screening and retaining the core peptide markers with stable diagnostic efficacy, constructing a more robust simplified diagnostic model, and evaluating its application potential in primary screening at the grassroots level; Fourthly, integrating serum peptide markers with conventional clinical indicators to construct a multi-dimensional combined diagnostic model, enhancing the comprehensive diagnostic value of the model; In addition, subsequent studies will also conduct in-depth exploration of the molecular mechanism of the core peptide markers in the occurrence and development of thyroid cancer through cell and animal experiments, providing potential targets for targeted therapy, and expanding the scientific connotation and clinical translation prospects of the research.

## 5 Conclusion

In summary, this study integrated serum proteomics data with eight machine learning algorithms to develop a diagnostic model for thyroid cancer based on multi-peptide features. The model demonstrated robust and superior diagnostic performance across multiple evaluation metrics, indicating strong disease identification capability and clinical translation potential. Functional analyses revealed that the identified proteins are involved in key pathways related to thyroid cancer development. These findings support the feasibility of non-invasive early diagnosis and provide a basis for further mechanistic and therapeutic studies.

## Data Availability

The original contributions presented in the study are included in the article/**Supplementary Material**. Further inquiries can be directed to the corresponding author.
