# Supplementary material for: Enhanced early detection of thyroid cancer using ensemble machine learning and serum proteomics
Source: Front Oncol. 2026 Mar 25;16:1807894. doi: 10.3389/fonc.2026.1807894 (PMC13056858; doi:10.3389/fonc.2026.1807894)
Supplement: Supplementary file 1 [file DataSheet1.docx]

**Supplementary material 1-Core model reproducibility details**

**Random seed:** experiment set all the stationary random seed 42, avoid the influence of random factors on experimental results, all of the code are available based on the seed repetition consistent results.

**Evaluation indexes:** the experiment USES 5 fold layered cross validation to evaluate training set and test set respectively, including the classification accuracy, precision ratio and recall ratio, F1 value, AUC - ROC, the mAP. Meanwhile, the experiment also visualized auxiliary evaluation graphs such as the ROC curve, PR curve, confusion matrix, learning curve, and calibration curve.

**Core super model parameters:** the experiment building eight kind of classical machine learning classification model, the core super model parameters are the actual use of a fixed value in the experiments, the specific as follows: K neighbor (KNN) : number of neighbor n_neighbors = 5, parallel computing n_jobs = 1; The kernel for kernel function of support vector machine (SVM) : = 'RBF', enable the probability forecast aim-listed probability = True; DecisionTree: Maximum depth max_depth=10; Gaussian Naive Bayes (GaussianNB) : Uses the default optimal parameters (no additional parameter adjustments); Adaptive Boosting (AdaBoost) : The base learner is a decision tree of depth 1, the number of iterations n_estimators=40, and the learning rate is set to default. RandomForest: The number of decision trees n_estimators=100, the maximum depth of a single tree max_depth=10, and the number of parallelism n_jobs=1; Lightweight Gradient Boosting (LGBM) The learning rate is learning_rate=0.05, the boosting type is gbdt, the objective function is binary, the number of leaves is num_leaves=31, the feature sampling rate is 0.8, the sample sampling rate is 0.8, both L1/L2 regularization are 0.1, and the sampling frequency is bagging_freq=3. Extreme gradient Boost (XGBoost) : Objective function binary:logistic, evaluation metric auc, iteration number n_estimators=100, maximum depth max_depth=5, learning rate learning_rate=0.05.

**Training configuration:** the training of the present study configuration includes standardized data pretreatment process and the rigorous model training process: In the data preprocessing stage, the classification labels "Mild ALI" are first mapped to 0 and "Severe ALI" to 1, and the invalid labels that have not been mapped are eliminated. Then, StandardScaler is used to perform standardization processing on all features with a mean of 0 anda variance of 1. Finally, the training set and the test set are hierarchically divided in an 8:2 ratio. The stratify parameter is used to ensure that the category distribution of the divided data set is consistent with the original data. The core configuration of the model training is to conduct model validation using stratified 5-fold cross-validation (StratifiedKFold) to ensure that the category distribution of each fold sample remains consistent with the original data. All models are uniformly set with n_jobs=1 to standardize the parallel training strategy and avoid result bias caused by hardware differences. At the same time, the principle of separating the training and testing processes is strictly followed. After completing the model training on the complete training set, the final performance evaluation is carried out on the independent test set, fundamentally avoiding the problem of data leakage.
